# Machine learning-based identification of small RNA signatures in aqueous humor as a step toward precision diagnosis of glaucoma

**DOI:** 10.1080/07853890.2025.2568119

**Published:** 2025-10-06

**Authors:** Margarita Dobrzycka, Anetta Sulewska, Joanna Konopinska, Piotr Karabowicz, Angelika Charkiewicz, Kinga Golaszewska, Mateusz Cedro, Przemysław Biecek, Jacek Niklinski, Alicja Charkiewicz, Radosław Charkiewicz

**Affiliations:** a Department of Ophthalmology, Medical University of Bialystok, Bialystok, Poland; b Department of Clinical Molecular Biology, Medical University of Bialystok, Bialystok, Poland; ;; c University of Antwerp, Antwerp, Belgium; d Faculty of Mathematics and Information Science, Warsaw University of Technology, Warsaw, Poland; e Department of Analysis and Bioanalysis of Medicines, Medical University of Bialystok; f Center of Experimental Medicine, Medical University of Bialystok, Bialystok, Poland

**Keywords:** Glaucoma, intraocular pressure, machine learning, neurodegenerative disease, retinal ganglion cell

## Abstract

**Background:**

Glaucoma is a progressive neurodegenerative disease of the optic nerve and one of the leading causes of irreversible blindness worldwide. Small RNAs (including miRNAs) play an important role in the pathogenesis of the disease. Despite extensive research, the exact mechanisms underlying glaucoma remain incompletely understood and current diagnostic tools primarily detect the disease at later stages, when substantial neuronal loss has already occurred. This study aimed to comprehensively analyze small RNA expression in the aqueous humor of patients with glaucomatous disease and identify specific RNA biomarkers associated with disease progression.

**Materials and Methods:**

This prospective, single-center, cross-sectional study involved 51 individuals: 31 adult patients with primary open-angle glaucoma (POAG) and 20 patients with cataracts qualified for elective surgery. A gradient-boosting decision tree classifier was implemented to classify POAG from cataracts. The model performance was rigorously assessed during cross-validation using a suite of metrics, including receiver operating characteristic (ROC) curves, area under the ROC curve, accuracy, recall, sensitivity, F1 score, specificity, precision, and negative predictive value.

**Results:**

Expression of hsa-miR-21-5p was significantly higher in POAG than that in cataracts, indicating its potential as both a biomarker and therapeutic target. Moreover, miR-210-3p expression was associated with apoptosis occurrence in trabecular meshwork cells treated with TGF-β1.

**Conclusions:**

Our study highlights the diagnostic potential of small-RNAs for distinguishing POAG from cataracts by integrating molecular profiling with advanced bioinformatics approaches. These findings provide new avenues for early diagnosis, personalized medicine, and improved clinical management.

## 1. Introduction

Glaucoma is a progressive neurodegenerative disease of the optic nerve and one of the leading causes of irreversible blindness worldwide. It is characterized by retinal ganglion cell (RGC) apoptosis, optic nerve head damage, and gradual loss of the visual field [1,2]. Despite extensive research, the exact mechanisms underlying glaucoma remain incompletely understood and current diagnostic tools primarily detect the disease at later stages, when substantial neuronal loss has already occurred. The most recognized risk factor for glaucoma is elevated intraocular pressure (IOP), which results from impaired aqueous humor (AH) outflow through the trabecular meshwork (TM) and Schlemm’s canal. However, IOP-independent mechanisms also contribute to disease pathogenesis, as some patients develop glaucomatous neuropathy despite having a normal or low IOP. This suggests that additional molecular regulators play important roles in disease onset and progression [3].

Over the past decade, the role of small RNA molecules, particularly microRNAs (miRNAs), in the pathophysiology of glaucoma has been highlighted [4–8]. MiRNAs are short, non-coding RNA molecules that regulate gene expression at the post-transcriptional level. They modulate key biological processes, including cell survival, inflammation, oxidative stress, extracellular matrix (ECM) remodeling, and neuroprotection. The dysregulation of specific miRNAs has been linked to glaucoma pathogenesis, particularly in relation to RGC apoptosis, TM dysfunction, and abnormal AH dynamics [6]. MiRNAs have been implicated in modulating the activity of the transforming growth factor-beta (TGF-β) pathway, which plays a considerable role in ECM deposition and fibrosis in the TM, contributing to increased outflow resistance and elevated IOP [9].

In addition to miRNAs, other small RNAs, such as circular RNAs (circRNAs), have been implicated in glaucoma. Studies have identified the differential expression of circRNAs in chronic ocular hypertension models; for example, *cZNF609* is upregulated in rat retinas and plays a role in glial activation [10]. Small-interfering RNAs have been experimentally used to silence glaucoma-related genes, such as *MRTF-B,* in TM cells, although their endogenous functions remain unclear [11]. PiRNAs and small-nucleolar RNAs are involved in glaucoma pathophysiology by regulating gene expression. However, direct evidence linking these small RNAs to glaucoma pathogenesis remains limited, highlighting the need for further investigation [12,13].

AH is a crucial ocular fluid involved in maintaining intraocular homeostasis. It provides an ideal medium for studying the molecular changes associated with glaucoma because it directly interacts with the ocular structures affected by the disease [14]. AH contains a variety of small RNA molecules, including miRNAs, which can serve as potential biomarkers for glaucoma. Unlike blood- or tear-based biomarkers, small RNAs in AH may offer a more specific reflection of intraocular pathological processes [15–17]. Several miRNAs, such as *hsa-miR-210-3p*, *hsa-miR-30a-3p*, and *hsa-miR-451a*, are differentially expressed in the AH of patients with glaucoma, suggesting their potential diagnostic and prognostic significance [16,18].

Despite these promising findings, research on small RNA biomarkers for AH remains limited. Most previous studies have focused on miRNA expression in the blood, which may not fully capture intraocular disease-specific alterations [16,19]. The functional roles of several glaucoma-associated miRNAs are poorly understood. Investigating small RNA profiles in AH could provide critical insights into the molecular mechanisms underlying glaucoma progression and therapeutic resistance. Additionally, identifying small RNA signatures associated with disease severity and treatment outcomes could improve risk stratification and tailor personalized treatment approaches.

This study aimed to comprehensively analyze small RNA expression in the AH of patients with glaucoma and identify specific RNA biomarkers associated with disease progression. By integrating molecular profiling with advanced bioinformatics approaches, this study aimed to enhance early glaucoma detection, refine prognostic models, and pave the way for novel therapeutic strategies that target small RNA-mediated regulatory mechanisms. The findings of this study have the potential to substantially advance the field of glaucoma research and provide new avenues for early diagnosis, personalized medicine, and improved clinical management of glaucoma.

## 2. Material and methods

This prospective, single-center, cross-sectional study enrolled 51 participants, including 31 adult patients diagnosed with primary open-angle glaucoma (POAG) (60.8%), eight of whom reported a family history of glaucoma (15.7%). Twenty patients with cataracts (39.2%) were also included, all qualifying for elective surgery. The study was conducted from April 2023 to April 2024 at the Ophthalmology Department and Department of Clinical Molecular Biology, Medical University of Bialystok (MUB), in accordance with the ethical standards stated in the Declaration of Helsinki. The research protocol was approved by the Bioethics Committee of MUB (number: APK.405.278.2022). Legally binding, conscious, written informed consent was obtained from all participants prior to their recruitment for the research. Indications for glaucoma surgery were as follows: patients with POAG in whom the target IOP level was not achieved despite maximally tolerated local and general IOP-lowering treatment, and documented progression of visual field defects, significant daily IOP fluctuations, or lack of patient compliance with the use of anti-glaucoma therapy or allergy to topical drugs. The indication for cataract surgery was clinically significant cataract classified according to the Lens Opacities Classification System (LOCS III (2) scale). The exclusion criteria for all groups were as follows: patients who had experienced a stroke or myocardial infarction in the past six months had a history of active cancer, had undergone ophthalmic surgery in the last six months, had acute eye inflammation or ocular trauma, or were monocular.

All patients underwent a detailed clinical examination, including a complete health and medication history. Ophthalmological evaluations included slit-lamp examination, IOP measurement using Goldmann applanation tonometry and fundoscopy (Table 1).

**Table 1.** Demographic characteristics of patients.

	POAG	CATARACT
Female	21 (67.7%)	12 (60.0%)
Male	10 (32.3%)	8 (40.0%)
Average age (years)	73	73
Average BCVA (Snellen chart)	0, 7	0, 6
Average IOP (mmHg)	19, 3	16
Average RNFL (μm)	63	99

Abbreviations: POAG, primary open angle glaucoma; BCVA, best corrected visual acuity; IOP, intraocular pressure; RNFL, retinal nerve fiber layer.

### 2.1. AH samples

AH specimens were obtained from patients scheduled for elective glaucoma or cataract surgery. Samples were intraoperatively collected through ab-externo limbal paracentesis using a 27-gauge needle connected to a tuberculin syringe. Stringent measures were implemented to avoid vascular disruption and safeguard against sample contamination. AH (50–200 µL) was extracted into a cryovial prior to the initiation of the principal corneal incision, followed by immediate storage in dry ice within the surgical suite. Specimens displaying signs of hemorrhage or failing to attain the minimum threshold of 50 µL were excluded from further evaluation. AH samples that were successfully harvested, preserved, and snap-frozen immediately after collection were conveyed in batches under rigorously controlled conditions (kept frozen on dry ice) to the Molecular Analysis Laboratory of the Department of Clinical Molecular Biology at MUB. The collected biological materials were analytically processed in grouped sets to maintain uniformity and precision.

### 2.2. RNA extraction

The AH specimens were gradually thawed on ice and subjected to centrifugation at 3000 × g for 15 min at 4 °C to pellet cellular components and debris. The supernatant was collected for RNA extraction. The supernatant volume was adjusted to 200 µL by the addition of a 10 mM Tris-HCl buffer (pH 8.0). RNA isolation was performed using a commercially available RNA purification system (miRNeasy Serum/Plasma Advanced Kit; Qiagen, Hilden, Germany), strictly adhering to protocols outlined by the manufacturer.

### 2.3. Library preparation and sequencing

Purified RNA extracted from each patient was used to construct sequencing libraries using the high-efficiency, low-input TrueQuant Small RNA-Seq Library Preparation Kit (GenXPro GmbH, Frankfurt, Germany) in strict accordance with the manufacturer’s instructions. Small RNA molecules were ligated to 3′ and 5′ adapters incorporating TrueQuant Unique Molecular Identifiers (UMIs; TrueQuant Adapters; GenXPro GmbH), followed by reverse transcription and polymerase chain reaction amplification using sample-specific index primers. Amplification was performed for a minimum number of cycles, followed by purification using magnetic beads. The resulting libraries were quality-assessed using a DNA analysis cartridge (Agilent High Sensitivity DNA Kit; Agilent Technologies, Santa Clara, CA, USA) on a microfluidics-based platform (Agilent Bioanalyzer 2100; Agilent Technologies). Subsequently, the validated libraries were pooled and subjected to paired-end sequencing with 2 × 65 bp reads on a next-generation sequencing platform (NextSeq 2000; Illumina, Inc., San Diego, CA, USA).

### 2.4. Sequencing data processing

Raw sequencing reads were subjected to adapter removal and quality filtering using Cutadapt (version 4.6) [20]. Deduplication was performed based on UMIs with a specialized software package (TrueQuant Software; GenXPro). Sequence quality was evaluated using FastQC (version 0.11.9; https://www.bioinformatics.babraham.ac.uk/projects/fastqc/). The processed reads were aligned to the hg38 (*Homo sapiens*) reference genome using Bowtie2 (version 2.4.4) [21] against multiple RNA databases, including miRNA (miRBase), transfer RNAs (tRNA; GtRNAdb), piRNA (piRNAdb), non-coding RNA (ENSEMBL), and complementary DNA (ENSEMBL). Alignment was conducted iteratively, wherein reads that were unmapped to a given reference were sequentially aligned to the subsequent database. Reads mapped to individual transcripts were quantified using the HTSeq software (version 2.0.2) [22]. A comprehensive summary report integrating the outputs from multiple analytical tools across all samples was generated using MultiQC (version 1.23) [23], facilitating the rapid detection of overarching trends and potential biases in the dataset.

### 2.5. Statistical and bioinformatics analyses

Differential expression analysis (DEA) was performed using DESeq2 (version 1.38) [24]. Log2FoldChange values were shrunk using “ashr” [25]. DEA results with a *p*-value ≤ 0.05 and an absolute log2FoldChange ≥1 were considered significant. Gene enrichment analysis of predicted targets for selected miRNAs in AH was executed using the DIANA-miRPath v4.0 platform [26]. This enrichment analysis evaluated the overrepresentation of miRNA target genes within specific Kyoto Encyclopedia of Genes and Genomes pathways.

To assess the diagnostic utility of the evaluated variables in differentiating POAG from cataracts, a gradient-boosting decision tree classifier was implemented. Across 100 randomized iterations, the samples were partitioned into training and test sets in a 3:7 ratio, with the class balance maintained within each subset. The model performance was rigorously assessed during cross-validation using a suite of metrics, including receiver operating characteristic (ROC) curves, area under the ROC curve (AUC), accuracy, recall, sensitivity, F1 score, specificity, precision, and negative predictive value. Results were reported as mean values with 95% confidence intervals. The established models were evaluated and analyzed using Shapley Additive exPlanations (SHAP) [27] to assess the impact of individual small RNA values on the model predictions. The model development and validation leveraged specialized Python libraries, specifically scikit-learn (version 1.0.2) [28,29], xgboost (version 1.7.6) [29,30], and shap (version 0.41.0) [27], to implement and optimize the gradient boosting decision tree classifier.

## 3. Results

### 3.1. DEA of small RNAs

**Figure 1.**
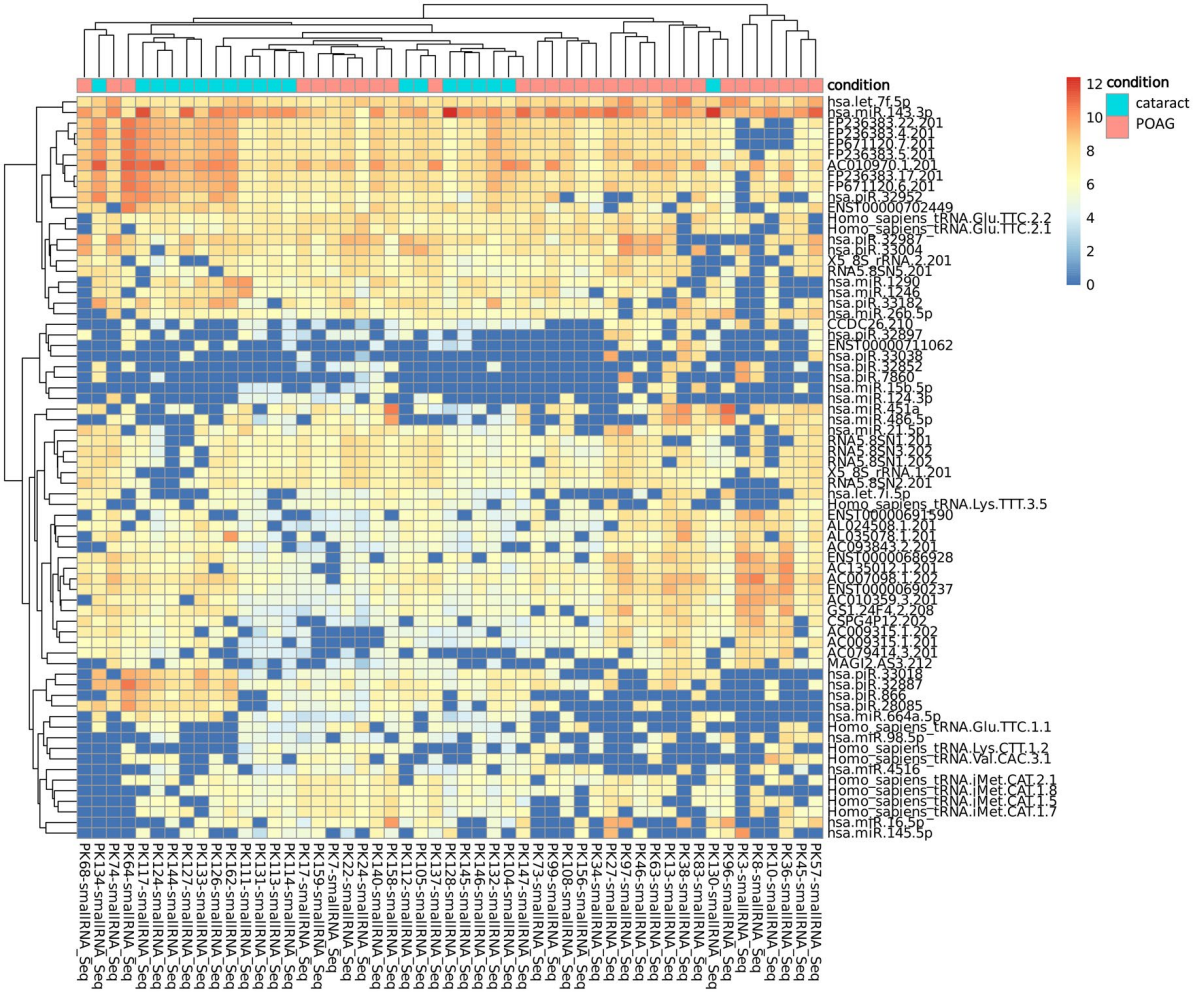
Heatmap illustrating expression patterns in POAG relative to cataract. Sample clustering was performed hierarchically using the pearson correlation coefficient, with molecular features curated according to the similarity of their expression signatures. Within the heatmap, high expression is represented by red hues, while blue hues depict low expression. Rows delineate individual differentially expressed entities, and columns correspond to distinct samples. POAG, primary open-angle glaucoma.

**Figure 2.**
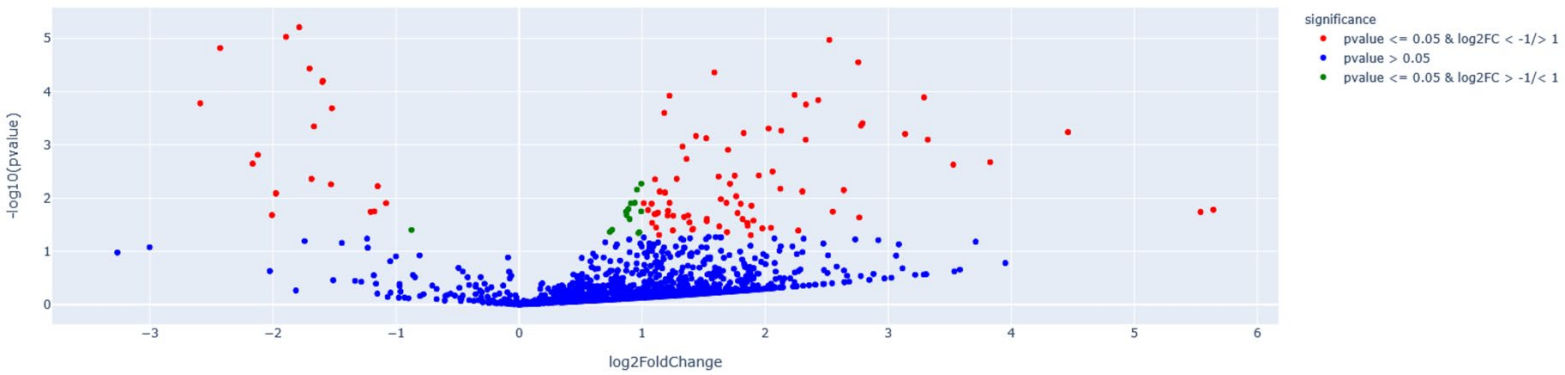
Volcano plot illustrating differential gene expression between POAG and cataract. The vertical axis displays the negative base-10 logarithm of p-values, while the horizontal axis quantifies the log_2_ fold change, calculated as the ratio of expression in the POAG group versus the cataract reference group. This graphical representation captures the interplay between statistical reliability and expression magnitude. Points corresponding to significantly upregulated molecular entities are positioned toward the right, whereas those markedly downregulated appear on the left. The uppermost region of the plot highlights features with the highest statistical significance, as indicated by the lowest p-values. Entities marked in red have a *p*-value ≤ 0.05 and log_2_FC ≤ −1 or ≥ 1; green have a *p*-value ≤ 0.05 and −1 < log_2_FC < 1; and in blue have a *p*-value > 0.05. POAG, primary open-angle glaucoma.

We commenced with a DEA of small RNAs to elucidate the molecular distinctions between POAG and cataracts. Ninety-two small RNAs were differentially expressed, comprising 20 miRNAs (21.7%), 13 piRNAs (14.1%), 24 long non-coding RNAs (lncRNAs) (26.1%), 16 tRNAs (17.4%), 10 ribosomal RNAs (10.9%), and 9 additional small RNA species (9.8%). Of these, 74 small RNAs (80.4%) were upregulated and 18 small RNAs (19.6%) were downregulated in POAG compared with those in cataracts. Comprehensive details pertaining to the attributes of all 92 identified small RNAs are presented in a heatmap (Figure 1), volcano plot (Figure 2), and Supplementary Table S1. A hierarchical clustering heatmap was constructed to graphically depict the expression disparities between POAG and cataract samples. This visualization confirmed the distinct molecular expression profiles for the two conditions, with elevated expression levels denoted in red and reduced expression levels denoted in blue. The resulting clustering patterns revealed pronounced segregation between the POAG and cataract groups, thereby reinforcing the molecular divergence associated with these pathologies. The small RNAs exhibiting the most pronounced differential expression (encompassing both upregulation and downregulation) in POAG relative to that in cataracts are detailed in Table 2.

**Table 2.** List of 40 small RNAs differentially expressed in POAG vs. cataract.

No. smallRNAs	log2FoldChange	*P*-value	FDR	Direction
hsa-piR-7860	5,64	0,02	0,09	upregulated
hsa-piR-33038	5,54	0,02	0,09	upregulated
hsa-piR-32852	4,46	<0,01	<0,01	upregulated
ENST00000711062	3,83	<0,01	0,02	upregulated
hsa-miR-15b-5p	3,53	<0,01	0,03	upregulated
hsa-miR-145-5p	3,32	<0,01	0,01	upregulated
hsa-miR-16-5p	3,29	<0,01	<0,01	upregulated
hsa-miR-486-5p	3,14	<0,01	<0,01	upregulated
AC079414.3-201	2,79	<0,01	<0,01	upregulated
hsa-miR-451a	2,78	<0,01	<0,01	upregulated
ENST00000691590	2,76	<0,01	<0,01	upregulated
hsa-piR-32897	2,64	<0,01	0,06	upregulated
hsa-miR-124-3p	2,55	0,02	0,09	upregulated
AC135012.1-201	2,52	<0,01	<0,01	upregulated
CSPG4P12-202	2,43	<0,01	<0,01	upregulated
AL035078.1-201	2,33	<0,01	<0,01	upregulated
AC009315.1-202	2,33	<0,01	0,01	upregulated
AC011453.1-201	2,27	0,04	0,16	upregulated
hsa-miR-4516	2,24	<0,01	<0,01	upregulated
ENST00000686928	2,13	<0,01	<0,01	upregulated
MAGI2-AS3-212	2,12	<0,01	0,05	upregulated
hsa-let-7i-5p	2,06	<0,01	0,03	upregulated
AC006480.2-201	2,05	0,04	0,15	upregulated
AC010359.3-201	2,03	<0,01	<0,01	upregulated
MORC2-AS1-201	1,98	0,04	0,15	upregulated
Homo_sapiens_tRNA-Lys-CTT-1-2	1,95	<0,01	0,04	upregulated
Homo_sapiens_tRNA-Val-CAC-3-1	1,89	0,01	0,08	upregulated
AC020978.7-201	1,86	0,03	0,14	upregulated
MT-RNR1-201	1,85	0,03	0,12	upregulated
AC007098.1-202	1,82	<0,01	<0,01	upregulated
Homo_sapiens_tRNA-Val-CAC-1-2	1,82	0,02	0,11	upregulated
hsa-miR-98-5p	1,80	0,01	0,08	upregulated
CCDC26-210	1,77	0,02	0,10	upregulated
Homo_sapiens_tRNA-Glu-TTC-1-1	1,76	<0,01	0,07	upregulated
GS1-24F4.2-208	1,75	<0,01	0,04	upregulated
AL024508.1-201	1,71	<0,01	0,05	upregulated
ENST00000690237	1,70	<0,01	0,02	upregulated
Homo_sapiens_tRNA-Val-TAC-1-2	1,69	0,04	0,16	upregulated
AC009315.1-201	1,69	0,01	0,08	upregulated
AC093843.2-201	1,64	0,01	0,07	upregulated
hsa-miR-92a-3p	1,63	0,03	0,14	upregulated
hsa-miR-21-5p	1,62	<0,01	0,04	upregulated
hsa-let-7f-5p	1,59	<0,01	<0,01	upregulated
RNA5S2-201	1,53	0,02	0,11	upregulated
AL031595.3-201	1,53	0,03	0,12	upregulated
RNA5-8SN5-201	1,52	<0,01	0,01	upregulated
5_8S_rRNA.2-201	1,44	<0,01	0,01	upregulated
Homo_sapiens_tRNA-Glu-TTC-4-2	1,41	0,04	0,15	upregulated
RNA5S5-201	1,41	0,04	0,15	upregulated
AL135790.1-201	1,37	0,02	0,10	upregulated
RNA5-8SN3-202	1,36	<0,01	0,02	upregulated
hsa-miR-3184-3p	1,34	0,02	0,10	upregulated
5_8S_rRNA.1-201	1,33	<0,01	0,01	upregulated
Homo_sapiens_tRNA-iMet-CAT-1-5	1,28	<0,01	0,04	upregulated
hsa-miR-101-3p	1,25	0,02	0,10	upregulated
Homo_sapiens_tRNA-Lys-CTT-4-1	1,25	0,04	0,16	upregulated
hsa-piR-32987	1,22	0,01	0,08	upregulated
Homo_sapiens_tRNA-Glu-TTC-2-2	1,22	<0,01	<0,01	upregulated
Homo_sapiens_tRNA-Lys-TTT-3-5	1,21	0,02	0,09	upregulated
Homo_sapiens_tRNA-Asp-GTC-3-1	1,21	0,02	0,10	upregulated
RNA5-8SN1-201	1,19	<0,01	0,06	upregulated
Homo_sapiens_tRNA-Glu-TTC-2-1	1,18	<0,01	<0,01	upregulated
RNA5-8SN2-201	1,14	<0,01	0,06	upregulated
RNA5S1-201	1,14	0,05	0,18	upregulated
hsa-piR-33004	1,13	0,02	0,10	upregulated
hsa-miR-26b-5p	1,12	0,02	0,10	upregulated
XACT-203	1,11	0,04	0,15	upregulated
Homo_sapiens_tRNA-iMet-CAT-1-8	1,10	<0,01	0,04	upregulated
hsa-piR-23127	1,10	0,02	0,10	upregulated
hsa-miR-100-5p	1,08	0,03	0,12	upregulated
RNA5-8SN1-202	1,08	0,01	0,08	upregulated
Homo_sapiens_tRNA-iMet-CAT-1-7	1,05	0,02	0,09	upregulated
Homo_sapiens_tRNA-iMet-CAT-2-1	1,01	0,01	0,08	upregulated
hsa-miR-143-3p	−1,08	0,01	0,08	downregulated
ENST00000702449	−1,15	<0,01	0,05	downregulated
hsa-miR-1290	−1,18	0,02	0,09	downregulated
hsa-miR-1246	−1,21	0,02	0,09	downregulated
FP236383.5-201	−1,52	<0,01	<0,01	downregulated
hsa-piR-33182	−1,53	<0,01	0,05	downregulated
FP236383.17-201	−1,60	<0,01	<0,01	downregulated
FP671120.6-201	−1,60	<0,01	<0,01	downregulated
AC010970.1-201	−1,67	<0,01	<0,01	downregulated
hsa-piR-32887	−1,69	<0,01	0,04	downregulated
FP671120.7-201	−1,70	<0,01	<0,01	downregulated
FP236383.22-201	−1,79	<0,01	<0,01	downregulated
FP236383.4-201	−1,89	<0,01	<0,01	downregulated
hsa-piR-28085	−1,98	<0,01	0,06	downregulated
Homo_sapiens_tRNA-Pro-TGG-1-1	−2,01	0,02	0,10	downregulated
hsa-miR-664a-5p	−2,12	<0,01	0,02	downregulated
hsa-piR-866	−2,17	<0,01	0,03	downregulated
hsa-piR-32952	−2,43	<0,01	<0,01	downregulated
hsa-piR-33018	−2,59	<0,01	<0,01	downregulated

### 3.2. Gene enrichment analysis (DIANA) of predicted targets for differentially expressed miRNAs in human AH

Of the 92 small RNAs identified in the DEA, 20 were miRNAs, which were selected for gene enrichment analysis using the DIANA-miRPath v 4.0 analysis platform [26]. This analysis enabled the identification of numerous biologically significant pathways associated with differentially expressed miRNAs (Table 3). Comprehensive results, including all enriched pathways, are provided in Supplementary Table S2.

**Table 3.** Gene enrichment analysis (DIANA) of predicted targets for the 20 most abundant small RNAs in human aqueous humor.

KEGG pathway	*P*-value	No. target genes	No. of small RNAs
Proteoglycans in cancer	1.05675E-13	130	19
Protein processing in endoplasmic reticulum	1.15071E-13	118	19
Autophagy—animal	8.28367E-12	92	18
Shigellosis	8.24253E-12	147	18
Cell cycle	2.55922E-11	83	19
Pathways in cancer	7.18942E-11	262	19
Prostate cancer	2.59625E-10	66	18
FoxO signaling pathway	2.54438E-10	84	19
p53 signaling pathway	2.60252E-10	53	18
Renal cell carcinoma	5.08375E-09	50	18
Ubiquitin mediated proteolysis	1.20628E-09	84	19
Spinocerebellar ataxia	1.79246E-09	85	18
Colorectal cancer	2.51637E-09	58	18
Phosphatidylinositol signaling system	1.28148E-08	63	18
Yersinia infection	1.32368E-08	84	18
Focal adhesion	2.25630E-08	112	19
Hippo signaling pathway	2.16827E-08	91	19
Salmonella infection	3.59691E-08	138	18
Bacterial invasion of epithelial cells	4.45184E-08	52	18
Adherens junction	8.88129E-07	51	18

### 3.3. Gradient boosting decision tree for assessing the diagnostic value of small RNAs in AH from patients with POAG

**Figure 3.**
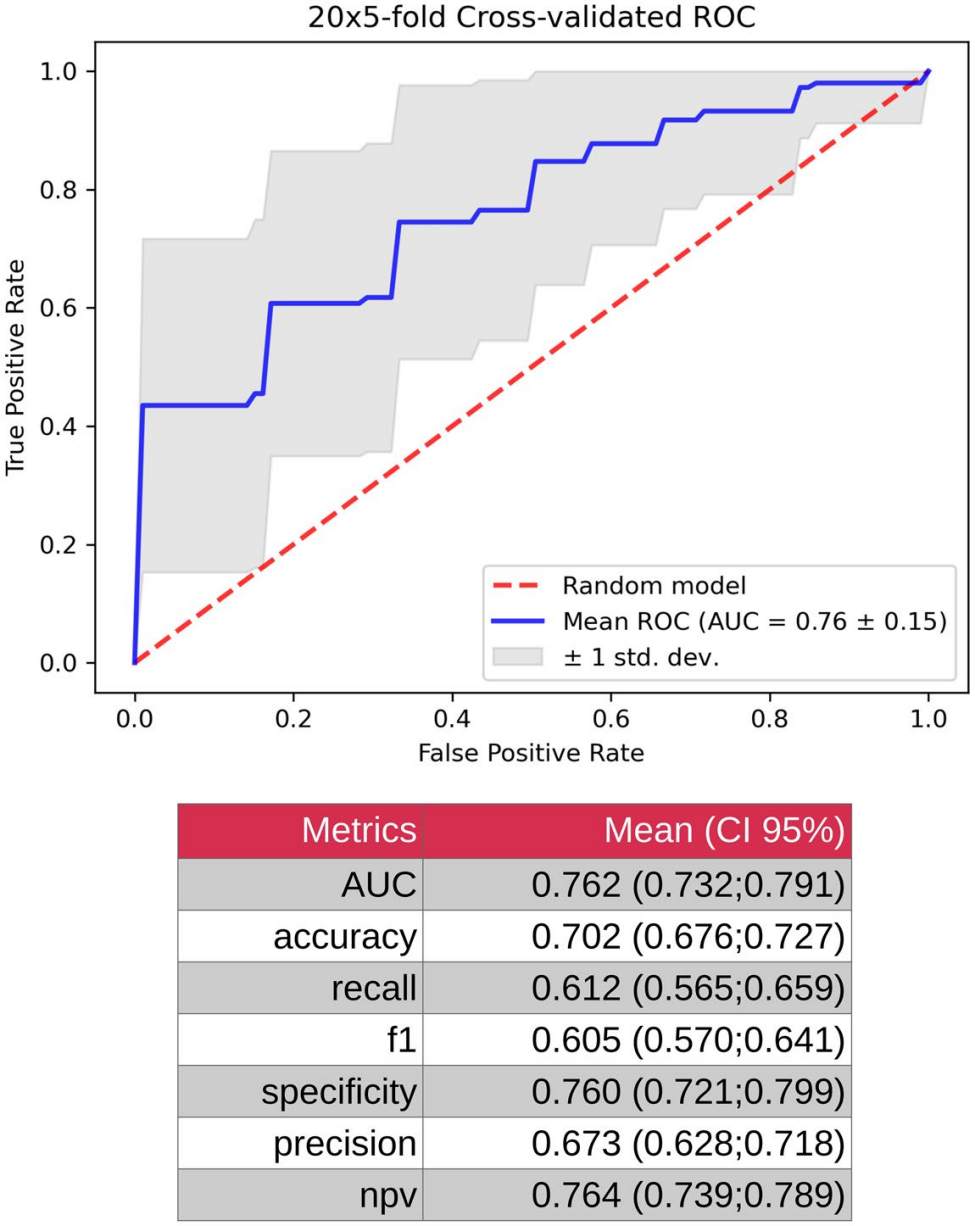
Model metrics for gradient boosting decision tree classifiers based on full set of 92 AH small RNAs. AH, aqueous humor.

To evaluate the diagnostic potential of small RNAs in distinguishing POAG from cataracts, normalized gene expression counts (transcripts per million) derived from DEA were used to construct a gradient-boosting decision tree classifier. This machine learning approach is a sophisticated and promising tool for advancing diagnostic applications in medical research. Initially, the classifier was trained using a full set of 92 AH small RNAs. The model exhibited moderate performance, achieving an AUC of 0.762, indicating a reasonable capacity to discriminate between POAG and cataracts. The accuracy was 70.2%, indicating that the model correctly classified approximately 70% of cases. However, the recall (sensitivity) was 61.2%, suggesting that the model was less effective in identifying true POAG cases and potentially overlooked some patients. The specificity, reflecting the ability to correctly identify cataract cases, was 76.0% (Figure 3).

**Figure 4.**
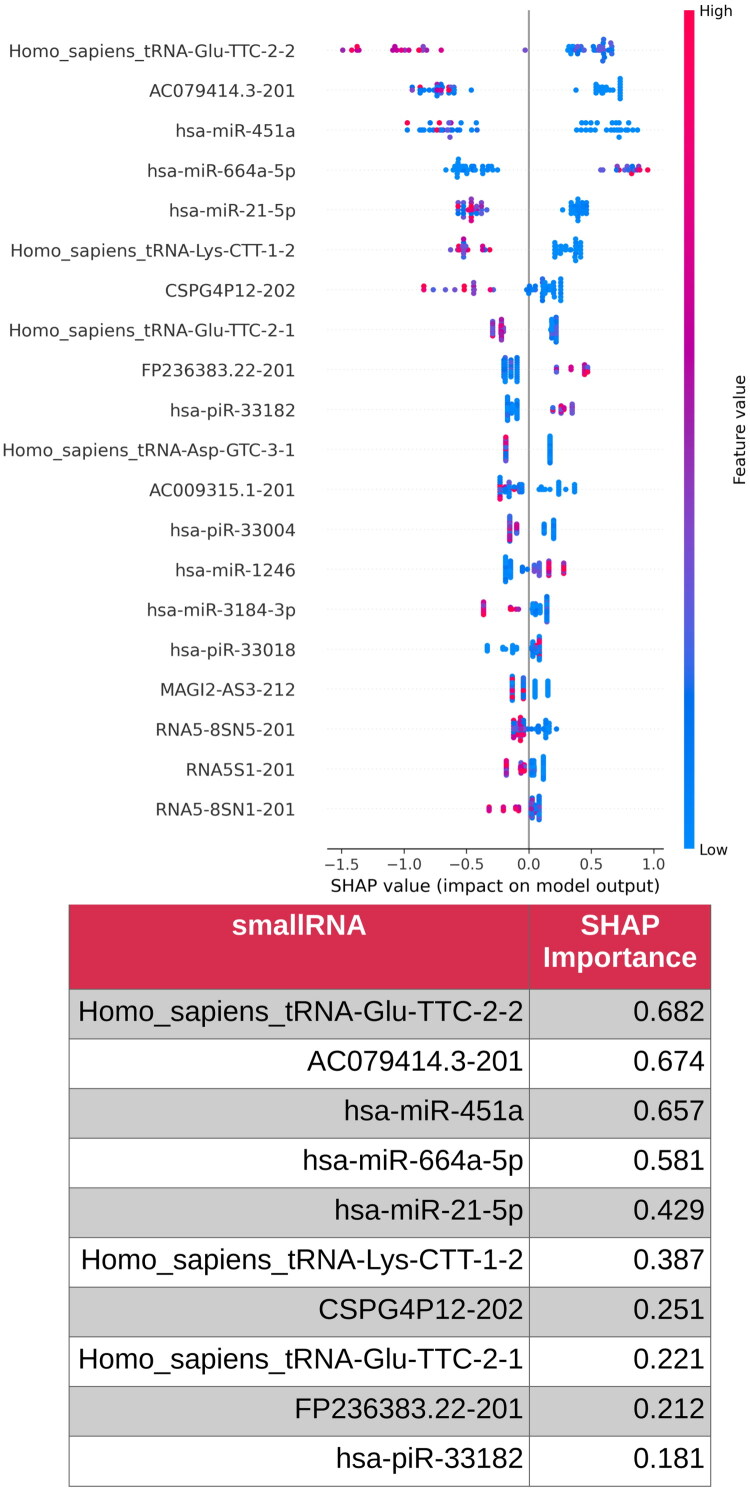
Shapley Additive Explanation for the gradient boosting decision tree classifier and 10 selected most important small RNAs.

The SHAP method was used to elucidate the contributions of individual small RNAs to the classification. This analysis identified 10 small RNAs as the most influential in differentiating POAG from cataract cases within the developed model (Figure 4). The top contributors were *Homo_sapiens_tRNA-Glu-TTC-2-2* (SHAP importance score: 0.682), *AC079414.3-201* (0.674), *hsa-miR-451a (0.657)*, *hsa-miR-664a-5p* (0.581), and *hsa-miR-21-5p* (0.429) (Figure 4). These molecules play significant roles in the classification process, highlighting their potential as molecular biomarkers for distinguishing POAG from cataracts.

**Figure 5.**
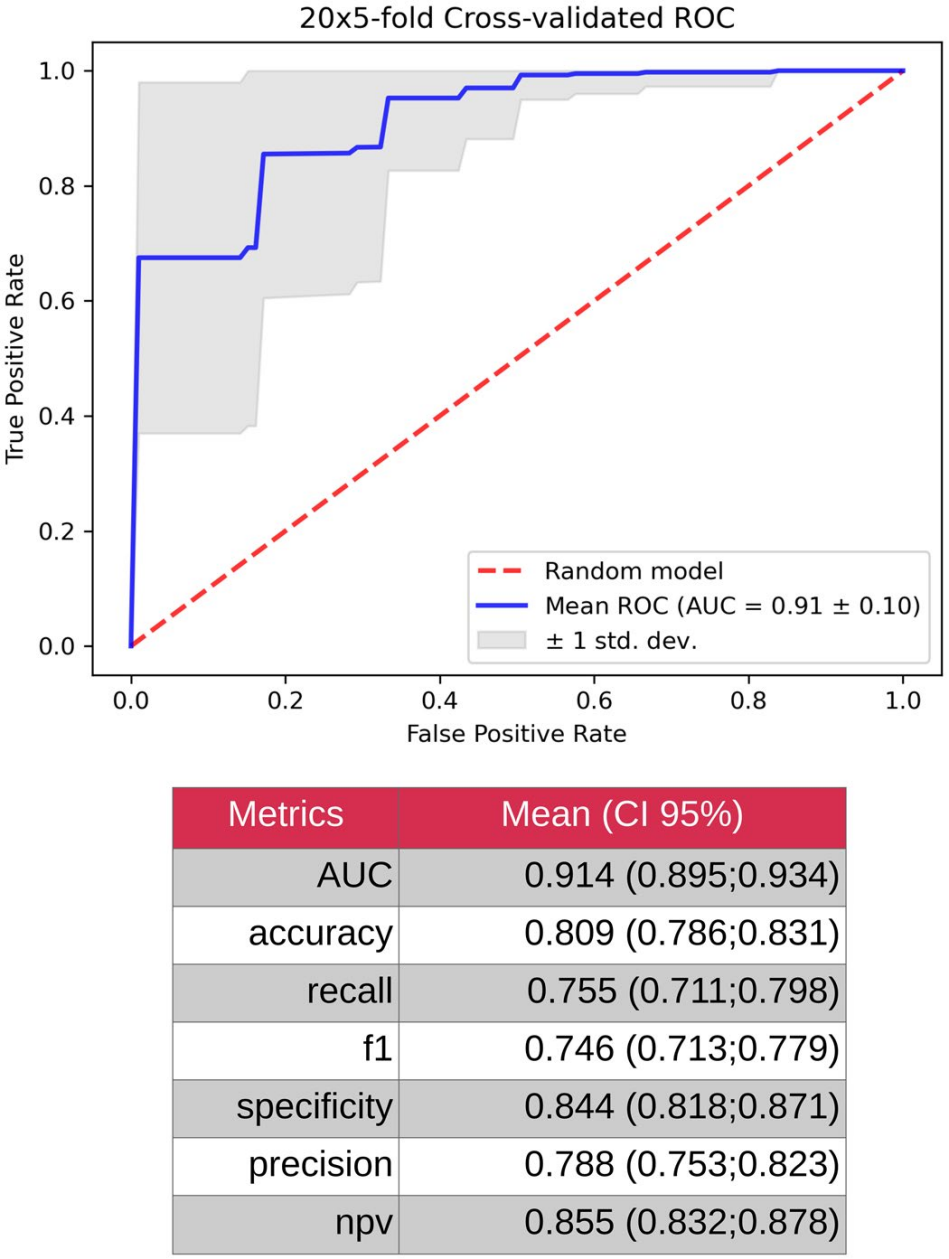
Model metrics for gradient boosting decision tree classifiers based on 10 small RNAs together.

Subsequently, the model was refined by restricting the feature set to the top 10 most impactful small RNAs identified using SHAP. This optimization significantly enhances the performance of the classifier. The AUC improved to 0.914, reflecting a substantially greater ability to differentiate between POAG and cataract cases. The accuracy increased to 80.9%, indicating an improved overall classification efficacy. The recall increased to 75.5%, reducing the number of missed POAG cases, and the specificity reached 84.4%, demonstrating enhanced accuracy in identifying cataract cases. These results collectively underscore that focusing on the 10 most relevant small RNAs significantly bolstered the discriminatory power of the model (Figure 5).

## 4. Discussion

This study demonstrates that small RNA molecules in the AH can effectively differentiate POAG from cataracts, as evidenced by the strong performance of our gradient-boosting decision tree classifier. Initially trained on 92 small RNAs, the model exhibited moderate discrimination, with an AUC of 0.762, accuracy of 0.702, specificity of 0.760, and recall of 0.612. By refining the model to include only the top 10 most significant genes, the classification performance significantly improved, reaching an AUC of 0.914, an accuracy of 0.809, a specificity of 0.844, and a recall of 0.755. This suggests that, within AH samples, a subset of small RNAs plays a particularly strong role in distinguishing glaucomatous changes from those observed in patients with cataracts.

Among these small RNAs, the lncRNA *AC079414.3-201* emerged as the second most influential feature, with a SHAP importance score of 0.674. As a transcript variant of an uncharacterized lncRNA [31], it is likely involved in gene regulation through epigenetic modifications, transcriptional control, or post-transcriptional interactions. Given the well-established role of oxidative stress in RGC apoptosis and TM dysfunction [32,33], the presence of *AC079414.3-201* in our high-performance model suggested its potential involvement in the modulation of these processes. Although its specific targets remain unknown, its strong association with disease classification warrants further investigation using RNA sequencing and functional studies. Similarly, another putative lncRNA, *FP236383.22-201*, was identified as an important feature that contributed to the improved classifier accuracy. LncRNAs often regulate chromatin remodeling and cellular homeostasis [34–36], therefore, FP236383.22-201 may play a role in TM function and IOP regulation, although further experimental validation is necessary to clarify its precise role in glaucoma pathology.

The pseudogene-derived transcript, *CSPG4P12-202* [37], related to *CSPG4*, a proteoglycan involved in ECM remodeling and neural development [38], is also among the most relevant features. Considering the importance of ECM homeostasis in TM function and optic nerve head integrity [39], it may subtly influence gene expression or miRNA availability in our model. Pseudogenes act as miRNA sponges, thereby regulating critical signaling pathways [40]. The high specificity of our refined model suggests that *CSPG4P12-202* contributes to distinguishing glaucoma from cataracts, although no direct functional evidence currently links this to glaucoma pathogenesis.

Notably, three tRNAs, *tRNA-Glu-TTC-2-2*, *tRNA-Glu-TTC-2-1*, and *tRNA-Lys-CTT-1-2*, were among the strongest molecular features that contributed to disease classification. Although tRNAs primarily function in protein translation, their presence in our model suggests that subtle translational shifts may occur in response to glaucomatous stress. Given that glutamate excitotoxicity is a key driver of RGC apoptosis [41], these tRNAs may indirectly reflect alterations in protein homeostasis within RGCs or supporting glial cells. The improved AUC and accuracy of our refined model indicate that these molecules may capture relevant disease-associated molecular changes, although their genomic redundancy suggests that they may not have specialized roles in glaucoma.

Several miRNAs identified in our study have well-established roles in post-transcriptional gene regulation and are aligned with known glaucoma mechanisms. *hsa-miR-451a* emerged as one of the most significant features, with a SHAP importance of 0.657. This miRNA is processed independently of Dicer and regulates oxidative stress responses by targeting *YWHAZ, RAB14,* and *AKT* [42,43]. Given that chronic oxidative stress contributes to RGC loss [44], its presence in the AH and its strong association with our mode recall and negative predictive value suggest that it may capture protective molecular signatures. However, further validation is required. Another miRNA, *hsa-miR-664a-5p*, has also been identified as a key regulator of proliferation, apoptosis, and inflammation *via* targets, such as *SOX7* [45]. Although its function in glaucoma remains largely unexplored, its contribution to the high precision of the classifier suggests that it may be involved in stress-related apoptotic pathways in ocular tissues.

*Hsa-miR-21-5p*, one of the most well-characterized miRNAs in the literature, was strongly associated with the performance of our classifier. This miRNA represses *PTEN, PDCD4,* and *SMAD7*, thereby modulating fibrosis, inflammation, and cell survival [46–48]. Its upregulation in glaucomatous TM and AH, as previously reported in the literature [49], aligns with its role in promoting fibrosis and IOP elevation through the TGF-β pathway [50]. The strong contribution of *hsa-miR-21-5p* to the high F1 score and AUC of our model suggests that it plays an important role in glaucoma pathogenesis, reinforcing its potential as both a biomarker and therapeutic target.

Additionally, our model identified *hsa-piR-33182*, a piRNA, as an important feature for classification. PIWI-interacting RNAs (piRNAs) typically function in transposable element silencing and epigenetic regulation. However, their roles in somatic cells remain poorly understood [51]. The inclusion of *hsa-piR-33182* in our model, particularly its contribution to high specificity, suggests that it may modulate oxidative stress or apoptotic pathways in the retina or TM. Although their precise function in glaucoma is unknown, their detection highlights the need for further research into the role of piRNAs in ocular diseases.

Hierarchical clustering analysis confirmed the distinct molecular expression patterns between POAG and cataract cases, supporting the findings of our classification model. Heatmap visualization demonstrates clear segregation of differentially expressed features, with high expression levels marked in red and low expression levels marked in blue. This clustering pattern further underscores the potential of these small RNAs as glaucoma biomarkers, reinforcing the biological relevance of our findings.

Gene enrichment analysis conducted using DIANA-miRPath v4.0 platform on the 20 most abundant miRNAs in the AH revealed key pathways linked to glaucoma pathogenesis. Significant enrichment in ‘“protein processing in endoplasmic reticulum” (*p* = 1.15 × 10^−13^) and “autophagy—animal” (*p* = 8.28 × 10^–12^) suggests roles in oxidative stress and RGC survival, while pathways, such as “cell cycle” (*p* = 2.56 × 10^–11^), “FoxO signaling” (*p* = 2.54 × 10^−10^), and “p53 signaling” (*p* = 2.60 × 10^−10^) indicate miRNA involvement in apoptosis and neurodegeneration. The enrichment of cancer-related pathways, including “proteoglycans in cancer” (*p* = 1.06 × 10^−13^) and “Hippo signaling” (*p* = 2.17 × 10^−8^), suggests a role in ECM remodeling and TM dysfunction, potentially affecting AH outflow. Additionally, immune-related pathways, such as “Shigellosis” (*p* = 8.24 × 10^–12^) and “Yersinia infection” (*p* = 1.32 × 10^−8^) highlight inflammation as a contributing factor, while the presence of “Spinocerebellar ataxia” (*p* = 1.79 × 10^−9^) aligns with the neurodegenerative aspects of glaucoma. These findings underscore the regulatory influence of miRNAs on glaucoma-related processes and suggest their potential as therapeutic targets.

This study highlights the potential of small RNAs in aqueous humor (AH) as biomarkers for distinguishing glaucoma from cataracts. However, several limitations must be acknowledged, along with strategies to address them. The relatively small and homogeneous cohort used in this study may limit the generalizability of the findings. This limitation reflects the inherent challenges of obtaining AH samples, given the invasive nature of the procedure, ethical considerations, and the restricted availability of patients undergoing surgeries that permit such sampling. These difficulties are well recognized in the field, with other studies frequently reporting even smaller cohorts (e.g. 26 and 18 patients) [17,52]. Despite these challenges, our study provides a strong foundation with high discovery potential, and future research will prioritize expanding cohort diversity and sample size to improve generalizability.

Another limitation is the cross-sectional design, which prevents conclusions about whether the identified small RNAs serve as early indicators of disease progression. To address this gap, we have initiated a longitudinal study that will follow a well-defined patient cohort, including early and advanced glaucoma cases, over a two- to three-year period. This study will integrate molecular, clinical, and imaging data and apply machine learning approaches to evaluate the predictive value of small RNAs for disease progression and intraocular pressure regulation.

In addition, AH samples may not fully reflect molecular alterations in primary glaucomatous tissues such as the retina and optic nerve head. To overcome this limitation, future studies will incorporate complementary biological sources, providing a more comprehensive assessment of disease-related pathways.

Finally, the functional roles of many differentially expressed small RNAs remain poorly characterized, and independent validation is essential. To ensure reproducibility, we plan to conduct qPCR-based validation in an independent cohort using standardized protocols. In parallel, mechanistic studies in cellular models, with extensions to animal models, will clarify the contribution of candidate small RNAs to intraocular pressure regulation and retinal ganglion cell survival. These investigations will bridge the gap between association and causation, reinforcing the clinical relevance of our findings.

Looking ahead, several strategies are being implemented to accelerate biomarker development and clinical translation. Large-scale discovery studies, such as ours, remain essential for identifying novel candidates, while validated biomarkers should be quantified using cost-effective and rapid assays such as quantitative PCR or digital droplet PCR. For panels involving more than six biomarkers, advanced techniques such as Hyperplex PCR can enable the simultaneous analysis of dozens of small RNAs. Integrating multi-omics approaches, including proteomics and metabolomics, will further enhance our understanding of glaucoma-related pathways, while non-invasive biomarker detection in blood or tear fluid may facilitate early diagnosis and improve patient compliance. Moreover, RNA-based therapies targeting key miRNAs or lncRNAs offer promising opportunities for therapeutic development.

By expanding cohort diversity, adopting longitudinal designs, validating findings across multiple tissues, and pursuing functional studies, future research can overcome current limitations and accelerate the translation of small RNA biomarkers into practical clinical tools. These efforts have the potential to transform glaucoma management by enabling earlier diagnosis, improving prognostic accuracy, and supporting personalized treatment strategies, ultimately reducing the global burden of this vision-threatening disease.

## 5. Conclusions

Our study highlights the diagnostic potential of small RNAs for distinguishing POAG from cataracts. The significant improvement in model performance after reducing the feature set to the most informative molecules demonstrated that specific small RNAs play a critical role in glaucoma pathogenesis. Identification of miRNAs, lncRNAs, pseudogene-derived transcripts, tRNAs, and piRNAs suggests that multiple regulatory layers contribute to disease progression, including oxidative stress, ECM remodeling, fibrosis, and translational shifts. Although miRNAs, such as *hsa-miR-21-5p* and *hsa-miR-451a*, align well with established glaucoma mechanisms, the roles of lncRNAs, pseudogene-derived transcripts, and piRNAs remain largely uncharacterized, necessitating further validation through RNA sequencing and functional assays. Moreover, this study provides new clinical insights into how machine learning-based identification of small RNA signatures can enhance glaucoma risk assessment and lead to modification of treatment algorithms. This may offer a promising strategy for reducing the impact of genetic glaucoma risk and transform the glaucoma prevention and management landscape. Future studies should focus on elucidating the precise functions of these molecules in glaucoma as they hold significant potential for biomarker development and targeted therapeutic interventions.

## Supplementary Material

Supplementary Table 2 final.xlsx

Supplementary Table 1 final.xlsx

## Data Availability

All materials and information are available upon an e-mail request on corresponding author.

## List of abbreviations:

AH: aqueous humor
AUC: area under the curve
BCVA: best corrected visual acuity
circRNAs: circular RNA
DEA: differential expression analysis
ECM: extracellular matrix
IOP: intraocular pressure
lncRNA: long non-coding RNA
miRNA: microRNA
MUB: Medical University of Bialystok
piRNA: PIWI-interacting RNA
POAG: primary open-angle glaucoma
RGC: retinal ganglion cell
RNFL: retinal nerve fiber layer
ROC: receiver operating characteristic
SHAP: Shapley Additive exPlanations
TM: trabecular meshwork
UMI: Unique Molecular Identifier
